# Concept of Using 3D Printing for Production of Concrete–Plastic Columns with Unconventional Cross-Sections

**DOI:** 10.3390/ma14061565

**Published:** 2021-03-22

**Authors:** Jacek Katzer, Aneta Skoratko

**Affiliations:** Faculty of Geoengineering, University of Warmia and Mazury in Olsztyn, 10-720 Olsztyn, Poland; aneta.skoratko@uwm.edu.pl

**Keywords:** 3D printing, formwork, plastics, fractals, reinforcement

## Abstract

A concept of concrete–plastic columns was presented in the paper. As a proof of concept, a research program was conducted. Seven different cross-sections of columns formwork were 3D printed using plastic. The cross-sections represented three types of columns’ shapes: most common, rare, and impossible to be realized using traditional formworks (based on fractals). Prepared plastic formworks were filled with cement mortar playing the role of ordinary concrete. After 28 days of curing, the load–strain characteristics of all the concrete columns were tested. Achieved results were discussed. It was proven that concrete–plastic columns were characterized by quasi-plastic behavior while being ultimately destroyed. Columns with fractal-based cross-sections sustained the largest strains while maintaining a significant part of the maximum load. The achieved results proved that it is possible to completely omit traditional steel rebar-stirrup reinforcement. The future direction of needed research should cover larger columns and other concrete–plastic elements. Using fiber-reinforced concrete for the creation of concrete–plastic elements should be also tested.

## 1. Introduction

Nowadays, 3D printing has become a technology that offers many opportunities in multiple industries including construction, mechanical, and biomedical engineering [1,2]. This additive manufacturing technology is based on the process of creating objects layer by layer [3,4]. The 3D printed objects are usually characterized by high accuracy, as they are created directly from a digital (e.g., CAD software) design [5]. 

Three-dimensional (3D) printed elements are also proposed as unconventional spatial reinforcement for concrete elements. The shape is selected on the basis of the desire to obtain stiffness and load-bearing capacity [6]. Unprecedented freedom of form, speed of erection of a structure, and reduced costs speak in favor of replacing traditional construction methods by 3D printing [7]. Some researchers even predict harnessing 3D printing for erection of Lunar and Martian outposts [8]. Currently erected structural concrete elements are often limited by the cross-section shape due to technological barriers (e.g., associated with the possibility of preparing formworks). Columns and pillars are good examples of such elements. They are present in the majority of erected buildings (especially of public functions) as structural supports or decorative elements. These columns and pillars have circular, square, or rectangular cross-sections [9]. Other geometrical shapes of columns cross-sections are possible to create using traditional formworks based on wooden planks. However, this approach is labor intensive. Traditional formworks based on wooden planks are currently almost fully replaced by shuttering systems, including lost (aka stay-in-place) formwork systems. The common adoption of shuttering systems by the construction industry significantly limited the practically possibility of achieving geometrical shapes of column cross-sections. In the authors’ opinion, harnessing 3D printing for the creation of column formworks would enable bypassing both the geometrical and labor barriers. The cross-section of 3D printed formwork could basically have any geometrical shape. 

Katzer and Szatkiewicz [10] proposed 3D printed plastic formwork for the creation of beams. The plastic formwork, after casting concrete, stays in place and plays the role of reinforcement of a concrete element. In this way, a significant reduction of total construction costs could be achieved (35–60%) by omitting the process of formwork demounting [11]. Other benefits associated with 3D printed plastic formworks are purely environmental and architectural. Three-dimensional (3D) printing has the potential to become a highly sustainable technology. Both filaments of natural and artificial origin can be used for printing. Currently, polymers (including polymers that are made from renewable resources) in the form of powder, resin, monomers, and thermoplastic fibers are used for 3D printing [12]. Plastic waste created during 3D printing (e.g., in the form of generated temporary supports, etc.) can be melted, transformed, and also recycled into new filaments. One may state that the technology of 3D printing is the key opportunity for preserving our natural environment [13,14]. Moreover, 3D printing offers total architectural freedom. Highly complicated geometrical shapes are possible to be cast using 3D printed plastic formworks and self-compacting concrete (SCC). Keeping all the above facts in mind, the authors decided to prove that it is possible to create concrete–plastic columns with unconventional cross-sections, characterized by quasi-plastic failure. During the research program, the column formworks were 3D printed and filled with self-compacting cement mortar, which played the role of concrete. The mortar was used instead of concrete due to the size of the 3D formworks. The formwork also played the role of reinforcement. Different cross-sections of columns were created, from very basic (square, circular) to highly complicated and impossible to be created by any traditional means (based on fractals of 3rd and 4th iteration). The prepared plastic–concrete column specimens were used for compressive strength tests. The full relation of load–strain was recorded up to ultimate destruction of a specimen. A quasi-plastic behavior of tested specimens was achieved. The conducted research proved that the idea of creating concrete–plastic columns with unconventional cross-sections characterized by quasi-plastic failure is feasible.

## 2. Materials and Design of Experiment

Standardized mortar described in EN 196-1 [15] was adopted as a brittle cement matrix. CEN (Comité européen de normalization) sand (EN 196-1) characterized by a median diameter [16] of 0.24 mm, cement CEM II 32.5R, and tap water were used to create the mortar. It was also modified by two admixtures: superplasticizer and stabilizer. Both admixtures were used to achieve very fluid and stable consistency of a fresh mix. The mixture composition of a mortar batch is presented in Table 1. Ingredients were dosed and mixed utilizing a mortar mixer and mixing procedure commonly used for the creation of standardized mortar commonly used for strength tests of cements [17]. 

Both superplasticizer and viscosity modifying agent were commercially available, and they fulfilled the requirements of BS EN 934-2:2009+A1:2012 [18]. The superplasticizer (commercially available as MC PowerFlow evo 508 (MC-Bauchemie, Środa Wielkopolska, Poland)) was based on polymerization technology. According to the producer, the superplasticizer is dedicated to be used for the production of ready-mixed concrete, precast elements, free-flowing, and self-compacting concretes. The viscosity modifying agent (commercially available as MC Centrament VMA 2 (MC-Bauchemie, Środa Wielkopolska, Poland) increases the cohesion within the cement paste, which reduces the fresh mix sedimentation and bleeding. As a result, high homogeneity can be achieved for free-flowing and self-compacting concretes. The achieved consistency of the fresh mortar mix was equal to 285 mm (determined by a flow table according to UNE EN 1015-3:2000/A2:2007 [19]). It was necessary to harness fresh mix characterized by such high flowability due to the complicated shape of some of the 3D printed formworks. Fresh mix had to be able to penetrate all formwork caverns without any mechanical compaction. Specimens used for tests of properties of mortar were in shape of prisms 40 mm × 40 mm × 160 mm and cubes 100 mm × 100 mm × 100 mm. Prisms were used for compressive strength tests and flexural strength tests. Cubes were used for tensile splitting test [20] and assessment of dynamic modulus of elasticity by means of an ultrasound method [21,22]. The mechanical characteristics of hardened mortar after 28 days of curing (in temperature of +20 ± 1 °C while being wrapped in polyethylene sheets) are presented in Table 2. 

The stay-in-place plastic formworks were created using commercially available 3D printer. The 3D printer was capable of printing different plastics, but acrylonitrile–co-butadiene–co-styrene (ABS) was chosen as a filament used during the research program. ABS is not a user friendly filament due to its tendency to delamination, possible after-printing shrinkage, and curling up at the ends of printed longer elements. Nevertheless, the filament proved to be very effective in case of robust 3D printed reinforcing elements for cement composites in previous research programs [6,10]. Its high mechanical properties together with the considerable resistance to chemical aggression were also a factor while choosing a filament type for the research program. The most important properties of the used ABS filament (as given by the producer) are presented in Table 3. 

**Figure 1:**
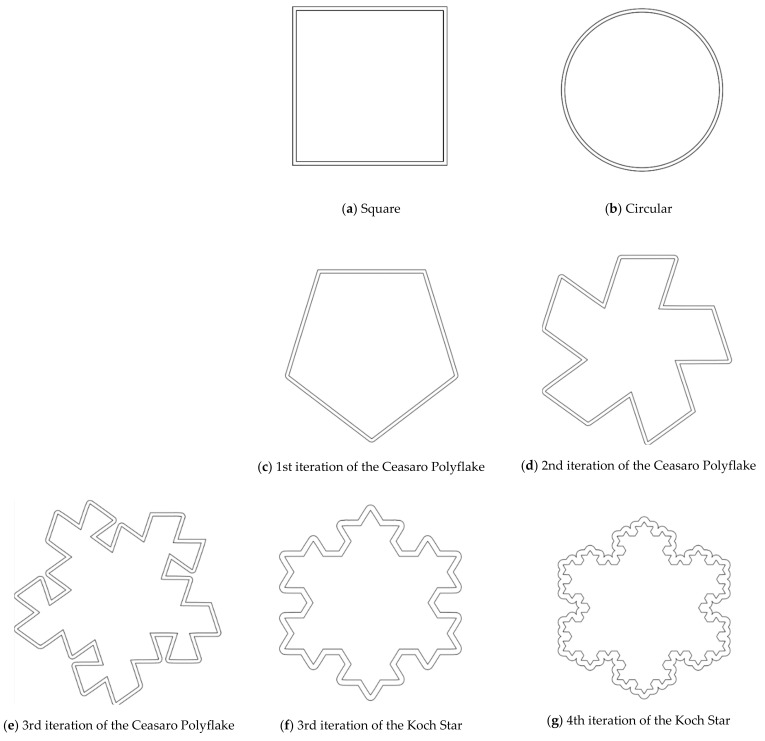
Cross-sections of 3D printed formworks.

The design of experiment covered three groups of plastic formwork cross-sections. The first group consisted of examples of cross-sections which are commonly used for the creation of columns or pillars. Formworks of circular and square cross-sections were allocated into this group. The second group consisted of examples of cross-sections that are possible to be created using traditional formwork techniques but would be very labor intensive to create. Formworks of a pentagon (first iteration of the Ceasaro Polyflake) cross-section and the second iteration of the Ceasaro Polyflake were allocated into this group. The third group consisted of examples of cross-sections which are impossible to be created with the help of traditional formwork techniques. Formworks with cross-sections based on more complicated fractals (namely: the third iteration of the Ceasaro Polyflake, the third and fourth iteration of the Koch Star) were allocated into this group. The shapes of all cross-sections in question are presented in Figure 1. It was decided that a prism 40 mm × 40 mm × 160 mm will play a role of a reference point in the research program. The prism (in an upright position) was a model of a concrete column with a cross-section area of 1600 mm^2^. All shapes of formwork cross-sections in question were designed in such a way that the area of cement mortar was always equal to 1600 mm^2^. The height of all 3D printed formworks was the same as the height of a prism specimen and equal to 160 mm. The thickness of formwork walls was equal to 1.6 mm. Using this research approach, one can state that all the differences in the mechanical characteristics of tested columns were caused by the shape of the formwork cross-section and subsequently by the amount of plastic used for its creation. 

**Figure 2:**
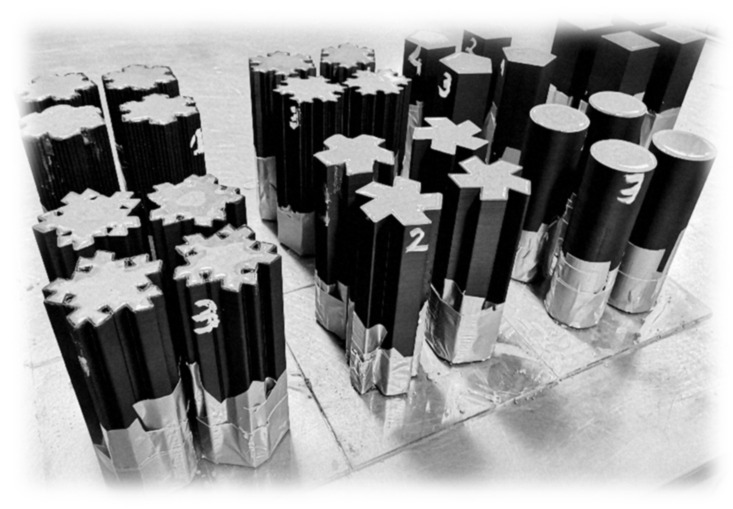
Cast concrete–plastic specimens.

The ABS stay-in-place formworks were 3D printed in upright positions in groups of four. The nozzle used for 3D printing was characterized by the diameter of 0.8 mm. Two layers of filament were needed to create each formwork wall. The average printing speed of 30 mm/s was utilized to maintain the integrity of printed slender formworks and the best possible quality of the print. Altogether, 28 formworks were 3D printed. The formworks were sealed by a duct-tape from the bottom and filled with the fresh cement mortar mix from the top. In Figure 2, 3D printed formworks just after filling with the fresh cement mortar mix are presented. The formworks were covered from top by glass sheets and left for 28 days for curing in temperature of +20 ± 1 °C. Before the tests, the duct-tape from the bottom parts of specimens was removed. 

## 3. Results

Specimens were tested after 28 days of curing using strength apparatus with the maximum loading force of 300 kN. The loading force was axial regarding the height of tested columns. The loading procedure consisted of the initial loading and proper loading. The initial loading of 100 N was kept for 30 s to let the specimens stabilize. Subsequently, the loading force was increased. The speed of loading was controlled by generated strains (0.5 mm/min). The loading was maintained until the ultimate destruction of a specimen or until reaching the strain of 5.6 mm (3.5% of the height of a specimen). 

**Figure 3:**
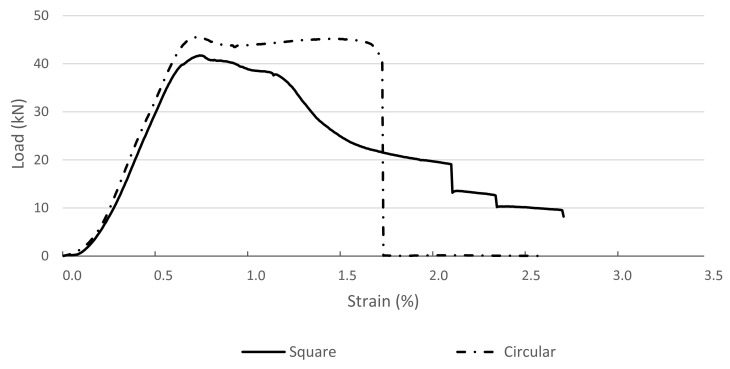
Load–strain relations for the tested columns of square and circular cross-sections.

**Figure 4:**
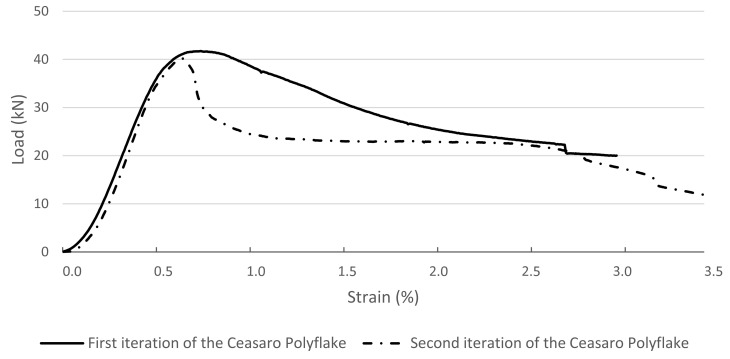
Load–strain relations for the tested columns with the cross-section in the shape of the first and second iteration of the Ceasaro Polyflake.

**Figure 5:**
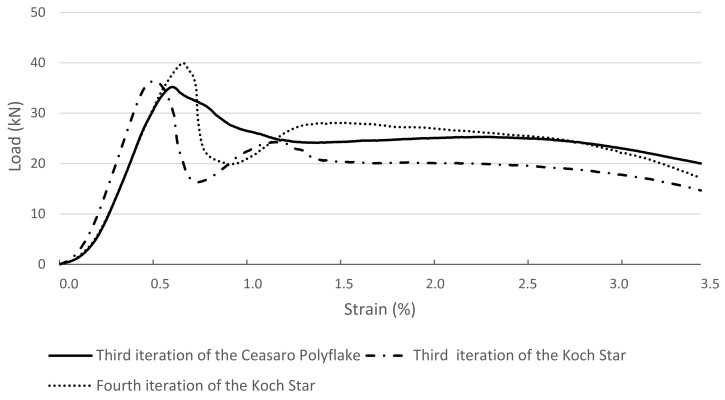
Load–strain relations for the tested columns with the cross-section in the shape of the third iteration of the Ceasaro Polyflake, third and fourth iteration of the Koch Star.

In Figure 3, Figure 4 and Figure 5, exemplary load–strain relationships registered during the tests are presented. The load–strain characteristics of cross-sections that are commonly used for the creation of columns or pillars (circular and square cross-sections) are presented in Figure 3. Both types of specimens reached the maximum loading force larger than 40 kN. The ultimate destruction of circular cross-section specimens was sudden and accompanied by breaking of the plastic stay-in-place formwork. Pieces of formwork were separated from the cement matrix, splintered, and some of them “burst” outside. The failure of the square cross-section specimens was less sudden, but the process of destruction was quite similar to the circular cross-section. No splinters of the destroyed formwork were “burst” outside in this case. In Figure 4, load–strain relationships are presented of specimens with cross-sections that can be created using traditional formwork techniques but would be very labor intensive to create. Both types of specimens are characterized by a very similar maximum loading force (of roughly 40 kN), but their behavior is much more quasi-plastic in comparison to the relations presented in Figure 3. Specimens reach two times larger strains before being ultimately destroyed. The destruction process is also accompanied by the breaking of the plastic stay-in-place formwork, but splintered pieces of formworks are not separated from the specimens. Sudden drops in value of loading force represent the destruction of a certain layer of 3D printed formwork. In case of a square cross-section, the process was started at the corners of a specimen, resulting in losing a layer of formwork. In case of a circular cross-section, the process was much more instant. The formwork was ultimately destroyed at almost the full height of a specimen. This phenomenon can be followed in load–strain relation (see Figure 3) when the loading force drops instantly from the value of over 40 to 0 kN.

**Figure 6:**
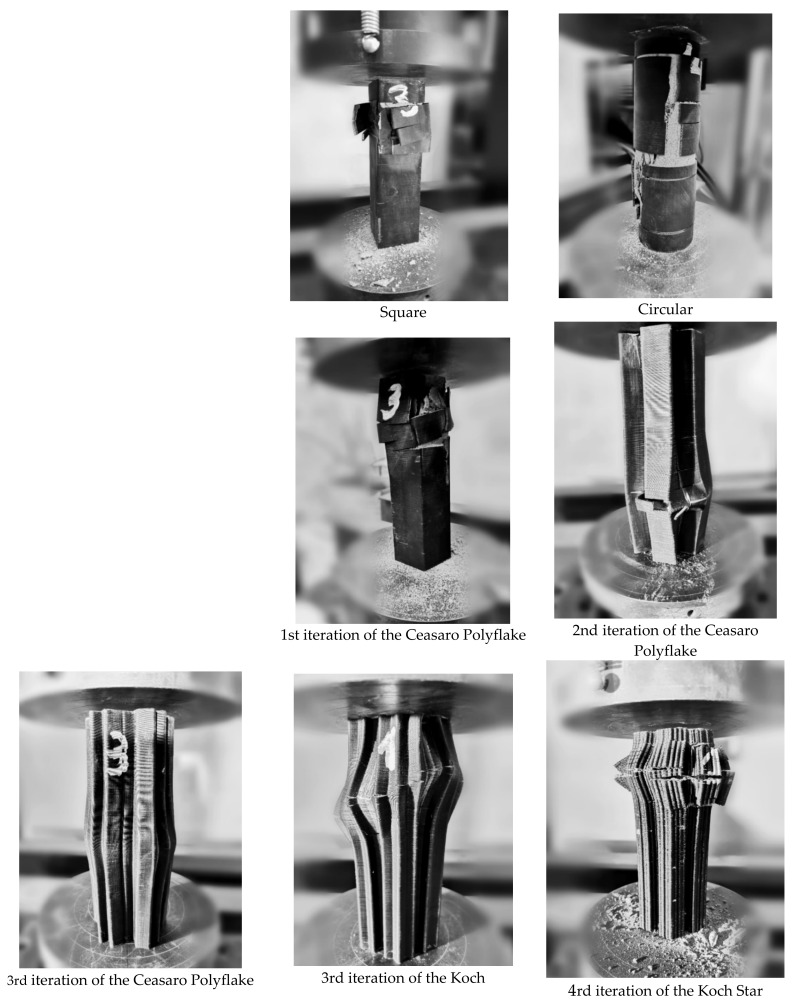
Images of specimens after loading.

In Figure 5, load–strain relations for cross-sections that are impossible to be created with the help of traditional formwork techniques are presented. Tested specimens with complicated cross-sections based on fractals (namely: the third iteration of the Ceasaro Polyflake, the third and fourth iteration of the Koch Star) are characterized by quasi-plastic characteristics. After reaching the highest loading force (from 35 to 40 kN), the subsequent destruction process is very smooth and associated with large strains. The shape of the load–strain curves resembles the behavior of fiber-reinforced concretes [23,24,25]. In all tested fractal specimens, the loading process was stopped after reaching the maximum planned strain of 3.5%. None of the specimens was ultimately destroyed at this point. Apart from vertical strains of the specimens, the loading process was associated with significant horizontal deformation of a cross-section. The plastic formwork did not break, but it was deformed, maintaining the quasi-plastic behavior of the whole specimen. Images of specimens after compressive strength tests are presented in Figure 6.

Registered load–strain relations enabled the calculation of the compressive strength (*f_C_*) of tested columns. For all specimens, the maximum achieved loading force (*F_MAX_*) was recognized and used for compressive strength calculations. Two versions of compressive strength were calculated, keeping in mind the specific geometric properties of 3D printed formworks (see Table 4). Version (a) of the calculated compressive strength (*f_C-CP_*) took into account both cross-sectional areas of plastic (*A_P_*) and concrete (*A_C_*) (full area of a cross-section). Version (b) of the calculated compressive strength (*f_C-C_*) took into account only the area of concrete. This approach was caused by a very low value of modulus of elasticity of the plastic filament in comparison to concrete. In the authors’ opinion, it is reasonable to assume that only the concrete core carries the axial compressive load, and the plastic formwork works only in tension, preventing the splitting of the concrete core. After reaching the *F_MAX_*, the concrete core finally splits, and its ultimate destruction is slowed down by the formwork. 

**Figure 7:**
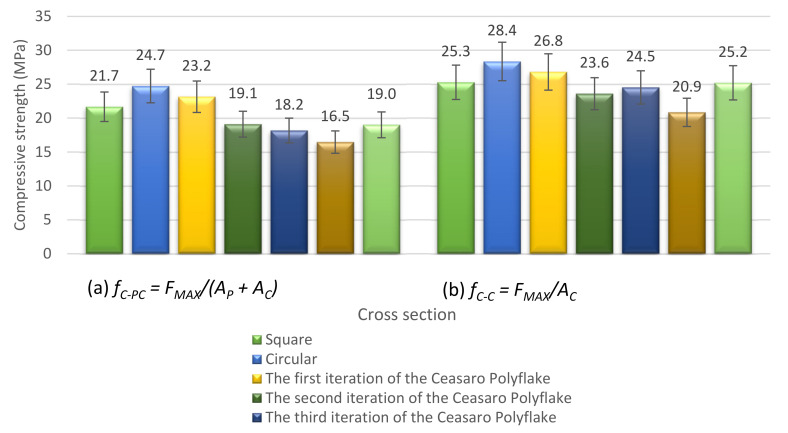
Compressive strength of tested concrete–plastic columns.

Both variants of calculated compressive strength are presented in Figure 7. One can notice that taking into account the area of plastic is especially important in case of fractal-based cross-sections. The area of plastic is the largest in these cases and can influence the calculated compressive strength by up to 6.3 MPa. 

## 4. Discussion

**Figure 8:**
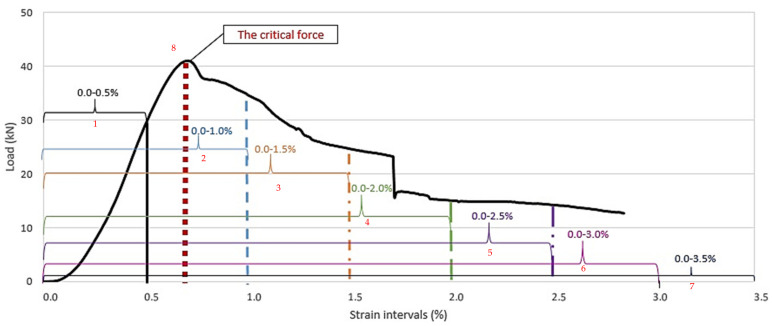
A scheme of strain intervals for which the energy was calculated.

Due to the non-conventional behavior of the tested columns with 3D printed plastic formwork (playing also the role of reinforcement), their compressive strength is not a sufficient parameter to capture their mechanical characteristics. Taking into account the shape of the achieved load–strain relations (which resemble relations achieved while testing fiber-reinforced concretes), the authors decided to use methods similar to those adopted in the technology of fiber-reinforced concretes [26]. For all tested specimens, the energy needed for their ultimate destruction was calculated. Namely, the area under the load–strain curve was computed in seven intervals associated with achieved strain (every 0.5%). The additional value of energy was calculated for the maximum load regardless at what value of strain it was reached. The graphic scheme of how the energy was calculated is presented in Figure 8.

**Figure 9:**
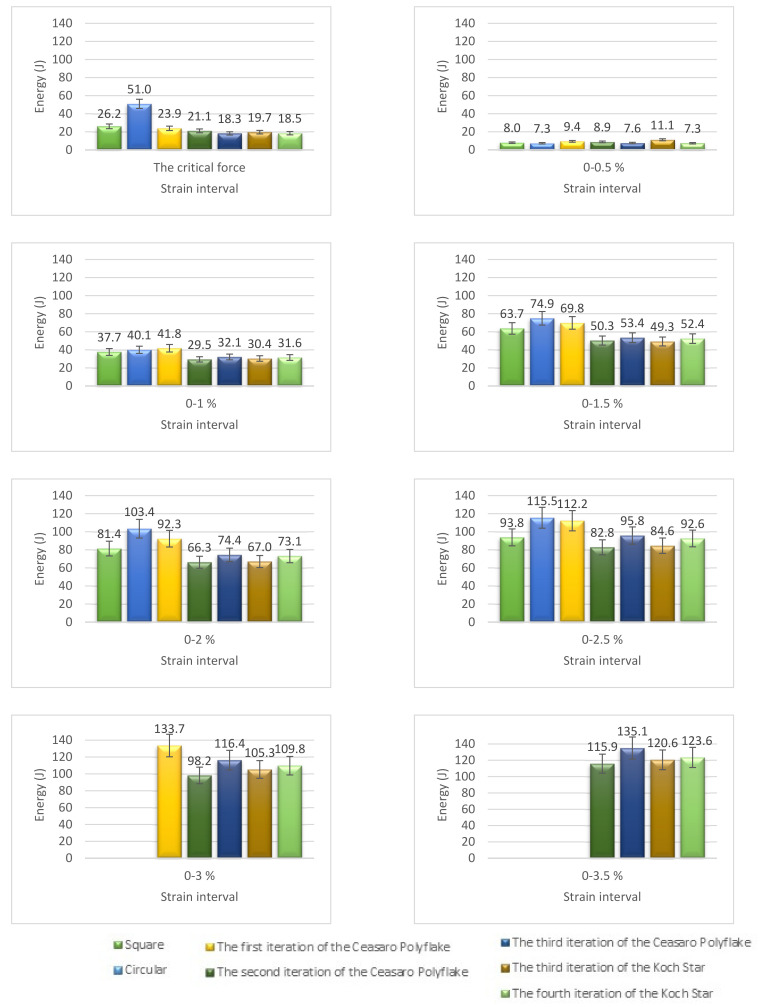
The energy needed to achieve a particular strain of tested concrete–plastic columns.

The energy needed to achieve particular strains of tested concrete–plastic columns is presented in Figure 9. Up to the strain of 2.5%, it was possible to register the energy for all tested specimens. For strains over 2.5%, the specimens with traditional (square and circular) cross-sections were destroyed; thus, the energy is not registered. Specimens with cross-sections that can be created using traditional formwork techniques but would be very labor intensive to create (1st and 2nd iteration of the Ceasaro Polyflake) are able to sustain a bit larger strains than specimens with square and circular cross-sections. In case of specimens with cross-sections that are impossible to be created with the help of traditional formwork techniques, the maximum value of strain planned to be realized was achieved, while specimens were still carrying significant load (50 ± 5% of the maximum load). The highest value of the registered energy was equal to 133.7 J and achieved by the column with the cross-section of the 1st iteration of Ceasaro Polyflake. This value is closely followed by energy values achieved by the 3rd and 4th iteration of Koch Star characterized by 120.6 J and 123.6 J, respectively, but registered at higher strains. From the traditional structural point of view, the most interesting are the values of energy needed to reach the maximum load. The circular cross-section proved to be the best solution at this stance with almost two times larger energy needed in comparison to the square cross-section and achieving the highest loading force and subsequently compressive strength. 

Fractal-based concrete–plastic cross-sections can not compete with circular and square cross-sections from a static point of view, but they provide brand new characteristics that make them an interesting option for the construction industry. 

The first novelty is associated with the automation of construction works. Three-dimensional (3D) printing is rapidly entering the construction industry, and in the near future, the erection of concrete–plastic columns with basic and very complicated cross-sections will be equally easy. Therefore, new architectural possibilities will be enabled. The conducted research program proved that it is possible to achieve columns with artistically pleasing shapes that are characterized by similar compressive strength to “boring” commonly erected circular and square cross-sections. 

The second novelty is associated with traditional reinforcement. Concrete elements (with no reinforcement) are brittle. The destruction process of such elements is sudden and ultimate. The tensile and flexural strengths of concrete in comparison to its compressive strength are not satisfactory. Therefore, almost all of the concrete used for structural purposes is reinforced by steel bars, stirrups, or less often by meshes or fiber [27,28,29,30]. Using plastic instead of steel reinforcement is a very tempting vision from the environmental point of view. Steel is expensive due to high amounts of energy needed for its production. At the same time, steel is fully recyclable. On the other hand, plastic is much cheaper and much more difficult to recycle than steel. It is possible to produce a plastic filament for a 3D printer from waste plastic [31,32,33,34,35]. The construction industry is able to utilize the vast amounts of recycled plastic in a form of printed formworks. As proved in the current research program and in a few previous ones [7,10], it is feasible to use 3D printed plastic elements for the reinforcement of concrete. 

The third novelty is associated with the quasi-plastic characteristic of a load–strain relation. Concrete–plastic columns (especially those with fractal-based cross-sections) are capable of absorbing high amounts of energy accompanied by large strains before being ultimately destroyed. Such behavior is not “employed” in the daily exploitation of a structure or building. Nevertheless, there are multiple scenarios in which such quasi-plastic characteristics are essential. The key scenarios are as follows: earthquake, explosion, accidental dynamic loading (e.g., lorry crash), and rapid flooding. The fractal-based concrete–plastic columns tested in this research program proved to have safe ultimate destruction behavior. While maintaining a significant percentage of the original compressive strength, they are being deformed in all three dimensions. During the ultimate loading, even at the maximum values of strains (equal to 3.5%), fractal specimens maintained their integrity (no plastic or concrete splinters were “burst”). Concrete–plastic columns after a destruction event (e.g., earthquake) would be significantly deformed but would enable the evacuation of people. Columns (and other concrete–plastic) structural elements after such an event would be commissioned for demolition, but plastic could be reused for a new filament and concrete rubble for the preparation of new coarse aggregate.

Architectural possibilities associated with almost unlimited shapes of created columns (and possibly other elements of a building) are overwhelming. One can forecast that complicated fractal based cross-sections of columns and surfaces of walls and ceilings, apart from purely artistic values, would have a significant influence on noise reduction in public spaces such as schools, train stations, airports etc. In the authors’ opinion, harnessing concrete–plastic elements for full-scale construction projects would create a brand new reality both in terms of engineering and architecture. Before achieving these goals, multiple problems should be addressed in future research programs such as low value of ABS modulus of elasticity, low resistance to fire, and aging of plastic associated with exposure to ultraviolet light. 

## 5. Conclusions

The conducted research program allows drawing the following conclusions:

- It is possible to create concrete–plastic columns with satisfactory mechanical characteristics,
- The proposed concrete–plastic columns enable the creation of columns with cross-sections that are impossible to achieve using traditional formwork solutions, thus opening brand new architectural and structural possibilities,
- Fractal-based columns have very safe characteristics of ultimate destruction, which should be useful during earthquake, explosion and other “emergency” loading scenarios,
- Circular and square columns allow reaching higher compressive strengths in comparison to fractal-based columns,
- More energy is needed to ultimately destroy fractal-based columns than to destroy circular and square columns,
- The proposed solution is associated with a significant increase in the automation of the construction process,
- Future research should cover larger concrete–plastic columns and concrete–plastic columns with the addition of fiber or with internal plastic spatial reinforcing elements,
- Other structural elements (e.g., beams, walls, slabs, etc.) should be considered for harnessing the proposed concrete–plastic solution with special interest in possible partial (or even full) elimination of traditional steel reinforcement.

## Figures and Tables

**Table 1:** Mixture composition of a mortar batch.

Ingredient	Amount (g)	Density (g/cm^3^)
Standardized sand	1350.0	2.65
Portland cement	450.0	3.10
Tap water	225.0	1.00
Superplasticizer	7.5	1.04
Viscosity admixture	7.5	1.01

**Table 2:** Mechanical characteristics of hardened mortar.

Property	Value	Unit
Compressive strength	22.7	MPa
Flexural strength	4.3	MPa
Splitting tensile strength	2.8	MPa
Apparent density	2040	kg/m^3^
Dynamic modulus of elasticity	32.0	GPa

**Table 3:** Key properties of the acrylonitrile–co-butadiene–co-styrene (ABS) filament.

Density	Melting Point	Diameter	Tensile Modulus	Tensile Stress at Break	Flexural Modulus	Flexural Strength
(kg/dm^3^)	(°C)	(mm)	(GPa)	(MPa)	(GPa)	(MPa)
1100	+225	2.85	1.7	33.9	2.1	70.5

**Table 4:** Geometrical characteristics of plastic formwork.

Shape of Specimen Cross-Section	Internal Circumference of the Formwork	Cross-Sectional Area of the Plastic of Formwork (*A_P_*)	Cross-Sectional Area of Concrete (*A_C_*)	Total Cross-Sectional Area(*A_P_* + *A_C_*)
(–)	(mm)	(mm^2^)	(mm^2^)	(mm^2^)
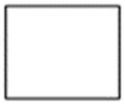	160.0	266.2	1600	1866.2
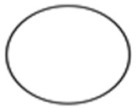	141.8	234.9		1834.9
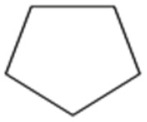	152.4	252.3		1852.3
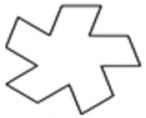	232.8	376.3		1976.3
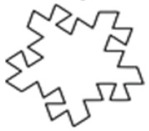	355.1	558.8		2158.8
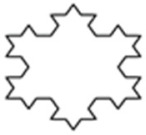	263.4	425.2		2025.2
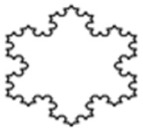	336.4	521.4		2121.4

## Data Availability

The data presented in this study are available on request from the corresponding author.
